# Factors influencing prolactin levels in chronic long-term hospitalized schizophrenic patients with co-morbid type 2 diabetes mellitus

**DOI:** 10.3389/fpsyt.2022.1034004

**Published:** 2022-10-18

**Authors:** Junhong Zhu, Huijuan Wang, Shaoyun Huang, Yingying Zhang, Xuebing Liu, Yi Li, Jun Ma

**Affiliations:** ^1^Department of Psychiatry, Wuhan Mental Health Center, Wuhan, China; ^2^Wuhan Hospital for Psychotherapy, Wuhan, China; ^3^Suzhou Guangji Hospital, Suzhou, China; ^4^Xinyang Vocational and Technical College, Xinyang, China

**Keywords:** long-term hospitalized, chronic, schizophrenia, diabetes mellitus, metabolic indicators, prolactin

## Abstract

**Background:**

For long-term hospitalized patients suffering from schizophrenia, metabolic disease and hyperprolactinemia (HPRL) are common comorbidities. This article is aimed at analyzing the factors influencing comorbid type 2 diabetes mellitus (T2DM) on prolactin (PRL) levels in long-term hospitalized patients suffering from schizophrenia.

**Methods:**

This study included 378 long-term hospitalized patients with schizophrenia. Common metabolic markers and PRL levels of included samples were collected, and the severity of psychopathology was assessed using the Positive and Negative Symptoms Scale (PANSS). Based on the patients with or without T2DM, the samples were divided into two groups. The differences in clinical parameters between the two groups were compared, and the effects of the parameters on the PRL levels were analyzed.

**Results:**

Compared with non-DM patients, the patients in the DM subgroup had lower PRL levels (*P <* 0.0001) and rather severe psychiatric symptoms (*P* = 0.016). Female, treated by risperidone, and high levels of triglyceride (TG) were faced with risk for HPRL (*B* = 26.31, *t* = 5.39, *P* < 0.0001; *B* = 19.52, *t* = 4.00, *P <* 0.0001*; B* = 2.71, *t* = 2.31, *P* = 0.022, respectively). Meanwhile, co-morbid DM and aripiprazole treatment were protective factors (*B* = 15.47, *t* = 3.05, *P* = 0.002; *B* = –23.77, *t* = –2.47, *P* = 0.014; respectively). Ultimately, in the DM subgroup, the dose of metformin was found to be a protective factor for HPRL (*B* = –0.01, *t* = –1.46, *P* = 0.047), while female and aripiprazole were risk factors (*B* = 16.06, *t* = 3.26, *P* = 0.001*; B* = 20.13, *t* = 2.57, *P* = 0.011*;* respectively).

**Conclusion:**

Aripiprazole is a protective factor for HPRL in long-term hospitalized patients, whereas the female is a risk factor. Metformin is beneficial in reducing PRL levels in patients with co-morbid DM. More aggressive and effective interventions are required for preventing adverse drug reactions in women and patients with co-DM.

## Introduction

Globally, disability-adjusted life-years (DALYs) for mental disorders increased from the 13th leading cause in 1990 to the 7th leading cause in 2019 (1). During this time, the age-standardized incidence rate (ASIR) and age-standardized DALYs rate (ASDR) of schizophrenia in China increased by 0.3 and 3.7%, respectively (2). According to data from China’s Guangdong Province, one of the reasons for the increased burden of schizophrenia in China is the large population base and aging population (3). Due to the unknown etiology and a lack of specific drugs, the cure rate for schizophrenia is low. According to a meta-analysis, approximately one-third of patients with first-episode schizophrenia were unresponsive or resistant to antipsychotic treatment (4). With the prolongation of the disease, the increase in the number of attacks, the randomization of prescription drugs, and other factors, the treatment response rate of schizophrenia becomes lower, and the response time becomes longer (5). Thus, many schizophrenic patients in China are indwelled in psychiatric hospitals for long-term in-patient treatment due to the aforementioned factors as well as due to reasons related to patients’ families and the loss of their working capacity caused by mental disability.

Metabolic syndrome is one of the common comorbidities of schizophrenia. Significant metabolic disturbances have been reported in schizophrenia, prior to the use of antipsychotics (6, 7). These metabolic disturbances are observed in approximately one-third of the total sick population (8). Furthermore, the widespread use of second-generation antipsychotics in recent years has had a negative impact on the metabolic syndrome of schizophrenic patients. Among them, two antipsychotics, clozapine, and olanzapine have rather prominent effects on metabolism (9). Diabetes mellitus (DM) is a globally prevalent metabolic disease. According to the International Diabetes Federation, about 537 million adults are currently living with DM, and the number is expected to increase to 643 million by 2030 (10). The need to manage metabolic comorbidities during hospitalization, particularly diabetes, is common in patients with schizophrenia predisposed to metabolic diseases. According to some studies, the comorbidity rate of severe mental illness and diabetes varies from 3.7 to 4.2% (11, 12). Another study from New Zealand reported that 41% of schizophrenic patients taking clozapine had abnormal blood sugar levels, and 26.48% suffered from co-DM (13).

Hyperprolactinemia (HPRL) is another common adverse reaction to antipsychotic drugs. The incidence of HPRL affects approximately 67–70% of patients taking antipsychotic drugs, depending on gender (14), and prolactin (PRL) levels were found to be affected dose-dependently with the use of antipsychotics (15). Although it has been suggested that the high levels of serum PRL caused by antipsychotic drugs can improve blood flow to some brain regions of patients, thereby acting as a neuroprotective agent (16, 17), a long-term HPRL status has a wide range of adverse physical effects, such as osteoporosis, male sexual dysfunction, breast development, female amenorrhea, gynecological tumors, etc. (18). Therefore, HPRL is regarded as one of the primary reasons for the decline in patient medication adherence.

Despite extensive and numerous studies on metabolic disorders and HPRL in schizophrenia, only a few studies have focused on the metabolic levels of long-term hospitalized schizophrenia patients and their relationship with PRL levels. Based on the huge population base of long-term hospitalized schizophrenic patients in China, the present research analyzed the factors affecting the PRL levels of long-term hospitalized patients, especially the patients with T2DM, in comparison with common metabolic indexes and PRL levels of co-T2DM and non-comorbid T2DM of schizophrenic patients. This study will provide suggestions and methods for the effective intervention of HPRL in this patient population.

## Materials and methods

### Subjects

A total of 378 patients with schizophrenia hospitalized for a long duration at Wuhan Mental Health Center and Suzhou Guangji Hospital from June 2018 to May 2019, were selected for this study.

#### Inclusion criteria

1. Meet the diagnostic criteria for schizophrenia mentioned in the International Classification of Diseases 10th Revision (ICD-10).
2. Age 18–70 years old, male or female.
3. The course of a psychiatric disorder is at least 6 years.
4. At least one-time point during the study period had 2 years of continuous hospitalization.
5. The total score of the Positive and Negative Symptom Scales (PANSS) was greater than 60 points.
7. There has been no antipsychotic adjustment in the last 3 months.
8. Type 2 diabetes mellitus (T2DM), hypertension, and hyperlipidemia were not excluded as common metabolic syndromes.

#### Exclusion criteria

Bipolar disorder, intellectual developmental disorder, dementia, severe depression, substance dependence, and other types of mental illnesses were excluded. Patients who use exogenous insulin (including those with T2DM requiring exogenous insulin for glycemic control and those with type 1 diabetes mellitus) and those with severe physical conditions other than the usual metabolic diseases that limit their capacity to carry out everyday tasks, such as severe heart disease, cerebral infarction sequelae, etc., were also excluded from the study. Additionally, patients with conditions including polycystic ovarian syndrome and PRL-secreting pituitary tumors that alter PRL levels were not included.

This study was reviewed and approved by the ethics committee of Wuhan Mental Health Center. The guardians of all participants knew about this study and signed informed consent.

### Research design

This study was designed as a cross-sectional study under natural observation conditions. The samples were divided into comorbid T2DM and non-T2DM groups based on whether the enrolled patients had T2DM. The differences in metabolic parameters between the two groups were compared, and the influencing factors of PRL levels in chronic long-term hospitalized patients with schizophrenia were analyzed. The influencing factors of PRL levels in subgroups of T2DM were further analyzed.

The PANSS assessment was completed on the same day for patients who met the inclusion criteria to determine the severity of the patient’s mental symptoms. The patient’s age, course of the disease, age of onset, duration of hospital stays, education level, and other information were extracted from the electronic medical record system. General clinical data such as body mass index (BMI), body weight (BW), abdominal circumference (AC), and type and dose of antipsychotic drugs in the last week (including the dose and type of hypoglycemic drugs), were extracted for patients with comorbid diabetes. The levels of fasting blood glucose (FBG), renal function, blood lipids, etc., detected for patients within the past month were also collected. For women of childbearing age with a regular menstrual cycle or taking antipsychotic medications that cause oligomenorrhea, the detection time of PRL was unified as the ovulation period of the patient’s menstrual cycle. The aforementioned parameters were entered into the self-created Excel spreadsheet program.

The PANSS evaluation was conducted in two sub-centers, each with two uniformly trained residents or attending physicians with more than 5 years of working experience.

### Data analysis

The mean and standard deviation of the normally distributed continuous measurement data were calculated, including the counts of the categorical variables. The independent sample *t*-test was used to compare data from different groups. The Chi-square test was used for the comparison of rates. The multiple linear regression model was constructed to analyze the influencing factors of PRL. All statistical tests were given a significance level of *P <* 0.05 (two tails).

## Results

### General clinical treatment characteristics

Table 1 presents the demographic and general clinical data of 378 patients with chronic schizophrenia in long-term hospitalization.

**TABLE 1:** The general clinical characteristics of the included patients.

Index	Included patients (*n* = 378)
Age—years	
Mean (SD)	48.12 (9.82)
Range	25–70
Gender—(n, %)	
Female	228, 60.32%
Male	150, 39.68%
Onset age—years	26.80 (8.97)
Course of disease—years	21.32 (9.34)
Length of hospital stay—years	4.56 (1.19)
Combined diabetes—(n, %)	
Yes	147, 38.89%
Metformin	72, 48.98%
Co-metformin	51, 34.69%
Others	24, 16.33%
No	231, 61.11%
Educational background—(n, %)	
High school and above	138, 36.51%
Middle school and below	240, 63.49%
Prescription drugs—(n, %)	
Clozapine	99, 26.19%
Combined with clozapine	96, 25.40%
Olanzapine	27, 7.14%
Risperidone	42, 11.11%
Quetiapine	27, 7.14%
Aripiprazole	15, 3.97%
Others	72, 19.05%

Metformin, metformin monotherapy for diabetes; Co-metformin, combined metformin for diabetes; Others, other drug regimens for diabetes.

### Differences in clinical parameters between schizophrenia patients with and without diabetes mellitus

The patients were divided into two groups based on whether they had comorbid DM, namely the group with DM (marked as group A) and the group without DM (marked as group B). The two groups were compared in terms of general clinical treatment, common metabolic parameters, and PRL (Table 2). Compared with group B, the course of disease, fasting blood glucose (FBG), uric acid (UA), triglyceride (TG), and PANSS scores were significantly increased in group A (*P <* 0.0001, *P* < 0.0001, *P* = 0.011, *P* < 0.0001, *P* = 0.016, respectively). Meanwhile, the onset age, blood uric acid (BC), high-density lipoprotein (HDL), low-density lipoprotein (LDL), and PRL were significantly decreased (*P* = 0.041, *P* = 0.001, *P* = 0.041, *P* = 0.021, *P* < 0.0001, respectively). Furthermore, the prescribed antipsychotic medications were significantly different between the two groups (*P* = 0.007).

**TABLE 2:** Differences in general clinical treatment and metabolic parameters between patients with diabetes and without diabetes.

Variable	Group A	Group B	*t*/χ^2^	*P*
Age—years	46.96 ± 10.20	48.86 ± 9.52	–1.84	0.067
Gender—(n, %)			2.50	0.114
Female	96, 65.31%	132, 57.14%		
Male	51, 34.69%	99, 42.86%		
Onset age—years	26.04 ± 9.35	27.98 ± 8.24	–2.05	0.041*
Course of disease—years	22.81 ± 8.50	18.98 ± 10.12	3.83	<0.0001*
Length of hospital stay—years	4.59 ± 1.24	4.55 ± 1.17	0.37	0.713
Educational background—(n, %)			0.01	0.967
High school and above	54, 36.73%	84, 36.80%		
Middle school and below	93, 63.27%	146, 63.20%		
Prescription drugs—(n, %)			17.61	0.007*
Clozapine	33, 22.45%	66, 28.57%		
Combined with clozapine	38, 25.85%	57, 24.68%		
Olanzapine	11, 7.48%	15, 6.49%		
Risperidone	12, 8.16%	30, 12.99%		
Quetiapine	15, 10.20%	12, 5.19%		
Aripiprazole	12, 8.16%	3, 1.30%		
Others	26, 17.69%	48, 20.78%		
BW—kg	60.60 ± 11.04	59.96 ± 10.49	0.57	0.569
AC—cm	80.24 ± 8.76	79.72 ± 8.73	0.56	0.579
BMI—kg/m^2^	22.75 ± 8.85	21.79 ± 3.44	1.48	0.139
FBG—mmol/L	7.33 ± 2.89	5.25 ± 1.00	10.02	<0.0001*
BUN—mmol/L	3.84 ± 3.06	3.79 ± 1.47	0.19	0.851
BC—mmol/L	63.89 ± 20.77	71.56 ± 21.06	–3.47	0.001*
UA—mmol/L	415.63 ± 121.80	385.31 ± 104.96	2.57	0.011*
TC—mmol/L	4.59 ± 1.11	4.69 ± 1.06	–0.89	0.375
TG—mmol/L	3.03 ± 2.38	2.08 ± 1.32	4.97	<0.0001*
HDL—mmol/L	0.99 ± 0.25	1.05 ± 0.28	–2.05	0.041*
LDL—mmol/L	2.24 ± 0.82	2.43 ± 0.80	–2.31	0.021*
PRL—ng/mL	31.27 ± 26.55	53.29 ± 49.53	–4.96	<0.0001*
PANSS	89.06 ± 11.11	86.17 ± 11.42	2.43	0.016*

Group A, schizophrenia comorbid diabetes; Group B, schizophrenia without diabetes.

BW, body weight; AC, abdominal circumference; BMI, body mass index; FBG, fasting blood glucose; BUN, blood urea nitrogen; BC, blood creatinine; UA, blood uric acid; TC, total cholesterol; TG, triglyceride; HDL, high-density lipoprotein; LDL, low-density lipoprotein; PRL, Prolactin; PANSS, Positive and Negative Symptom Scales.

*P < 0.05.

### Analysis of influencing factors of prolactin levels in included patients

Using PRL as a dependent variable, the following variables were used as independent variables: gender, onset age, course of the disease, DM (0 = co-comorbidity, 1 = non-comorbidity), quetiapine, olanzapine, aripiprazole, risperidone, clozapine, FBG, BC, UA, TG, LDL, and PANSS scores (all the antipsychotics involved were marked as default 0 = non-prescribed, 1 = prescribed). A multiple linear regression model was constructed, shown in Table 3. We found that female, risperidone, and high levels of TG were risk factors for HPRL (*B* = 26.31, *t* = 5.39, *P* < 0.0001; *B* = 19.52, *t* = 4.00, *P* < 0.0001*; B* = 2.71, *t* = 2.31, *P* = 0.022, respectively). Meanwhile, co-DM, aripiprazole, and high levels of UA were protective factors for HPRL (*B* = 15.47, *t* = 3.05, *P* = 0.002; *B* = –23.77, *t* = –2.47, *P* = 0.014*; B* = –0.05, *t* = –2.49, *P* = 0.013*;* respectively).

**TABLE 3:** Influencing factors of prolactin levels: Multiple linear regression model.

	B(SE)	95%CI	*t*	*P*
Constant	16.40 (26.18)	–35.08 to 67.88	0.63	0.531
Gender (male vs. female)	26.31 (4.88)	16.71–35.90	5.39	<0.0001*
Onset age—years	–0.24 (0.26)	–0.75 to 0.26	–0.96	0.339
Course of disease—years	–0.10 (0.26)	–0.61 to 0.41	–0.37	0.710
Diabetes mellitus	15.47 (5.07)	5.50–25.45	3.05	0.002*
Quetiapine	–10.99 (7.18)	–25.10 to 3.13	–1.53	0.127
Olanzapine	–0.73 (7.33)	–15.15 to 13.68	–0.10	0.920
Aripiprazole	–23.77 (9.64)	–42.72 to –4.82	–2.47	0.014*
Risperidone	19.52 (4.89)	9.92–29.13	4.00	<0.0001*
Clozapine	2.35 (5.39)	–8.25 to 12.95	0.44	0.663
FBG—mmol/L	–2.01 (1.06)	–4.09 to 0.07	–1.90	0.058
BC—mmol/L	0.07 (0.11)	–0.15 to 0.29	0.62	0.536
UA—mmol/L	–0.05 (0.02)	–0.09 to –0.01	–2.49	0.013*
TG—mmol/L	2.71 (1.18)	0.40–5.02	2.31	0.022*
LDL—mmol/L	1.13 (2.52)	–3.83–6.09	0.45	0.655
PANSS	–0.20 (0.18)	–0.55 to 0.16	–1.09	0.277

FBG, fasting blood glucose; BC, blood creatinine; UA, blood uric acid; TG, triglyceride; LDL, low-density lipoprotein; PANSS, Positive and Negative Symptom Scales.

*P < 0.05.

### Analysis of influencing factors of prolactin levels in schizophrenia patients with diabetes mellitus

Based on the above findings, we investigated the factors influencing PRL levels in schizophrenia patients suffering from DM (Table 4). Multiple linear regression models were constructed, using PRL levels as the dependent variable, whereas metformin dose, gender, aripiprazole, risperidone, UA, and TG were considered as independent variables (The antipsychotics involved were marked by 0 = non-prescribed, 1 = prescribed). Finally, we found that metformin was a protective factor for HPRL (*B* = –0.01, *t* = –1.46, *P* = 0.047), whereas female and aripiprazole were risk factors for HPRL (*B* = 16.06, *t* = 3.26, *P* = 0.001*; B* = 20.13, *t* = 2.57, *P* = 0.011, respectively).

**TABLE 4:** Influencing factors of prolactin levels in patients with diabetes mellitus: multiple linear regression model.

	B(SE)	95%CI	*t*	*P*
Constant	11.46 (14.05)	–16.32 to 39.24	0.82	0.416
Metformin dose—mg	–0.01 (0.00)	–0.01 to 0.00	–1.46	0.047*
Gender (male vs. female)	16.06 (4.93)	6.33–25.80	3.26	0.001*
Aripiprazole	20.13 (7.84)	4.63–35.63	2.57	0.011*
Risperidone	–7.28 (5.09)	–17.35 to 2.78	–1.43	0.155
UA—mmol/L	–0.501 (0.02)	–0.05 to 0.03	–0.61	0.541
TG—mmol/L	1.16 (1.00)	–0.82 to 3.14	1.16	0.249

UA, blood uric acid; TG, triglyceride.

*P < 0.05.

## Discussion

The present study reported that the schizophrenia group with DM had an earlier onset of mental illness, a longer overall psychiatric course, and more severe residual psychiatric symptoms under natural observation conditions. Other metabolic indicators (such as TG, UA, and HDL) were also poorly controlled, apart from poor control of blood sugar levels. Additionally, PRL levels in DM clinical subgroup were lower than in those without DM subgroup. When the factors affecting PRL in the included population were particularly focused, it was found that co-DM, prescription aripiprazole, and UA levels were the protective factors of PRL, whereas female, prescription risperidone, and high levels of TG were found to be functioning as risk factors. We further analyzed the influencing factors of PRL in the subgroup of comorbid DM and found that metformin dose and male were the protective factors of HPRL. However, the prescription aripiprazole lost its protective effect on HPRL and became a risk factor.

Antipsychotics induce HPRL by blocking dopamine D_2_ receptors on anterior pituitary PRL-releasing cells. Thus the release of PRL from these cells is no longer inhibited by dopamine and more PRL is secreted (19). Despite the reported studies, no specific intervention is required when antipsychotic-induced PRL levels are below 50 ng/mL (20). A guideline suggests that interventions are only needed for symptomatic antipsychotic-induced HPRL (18). However, some guidelines recommend more aggressive treatment and intervention for antipsychotic-induced HPRL, even without symptoms (21). Regardless, the negative effects of HPRL are well-defined and pervasive, necessitating aggressive intervention and meticulous management. Reducing drug dosages, switching antipsychotic drug types, co-prescribing dopamine agonists, and adding low-dose aripiprazole are typical therapies in psychiatric therapeutic care for HPRL, with the last having a greater level of evidence and recommendation (22). A further established safe and efficient strategy is the co-prescription of dimethicone (23).

We discovered in this study that patients with co-DM had more severe psychopathology, younger onset, and longer disease duration. This should indicate that patients are more likely to receive antipsychotic prescriptions earlier, for a longer duration, and in higher doses. It also indicates that patients are more likely to be metabolized by earlier prescriptions of clozapine, which has a more pronounced side effect (9). This might also contribute to the development of diabetes comorbidities like elevated UA levels and unfavorable lipid profiles. Some studies suggest that the dose of antipsychotics influences some adverse medication reactions (such as hyperglycemia, HPRL, weight gain, and so on) in schizophrenic patients (23). This research is based on chronic psychotic patients with a long course of the disease; thus, it is difficult to trace the types of antipsychotic drugs, they have been taking in their medical history. The types of antipsychotic drugs prescribed are also differing and complex; thus, association analysis of metabolic markers with the dose and exposure time of antipsychotics could not be performed. Only a portion of the qualitative analysis was carried out.

Aripiprazole, as a relatively unique antipsychotic drug, has the pharmacological effect of partially activating the D_2_ receptor, which has been demonstrated in numerous studies and is recognized by the industry to improve HPRL caused by antipsychotics. Simultaneously, aripiprazole has also been recommended in the guidelines for treating prolactinoma and HPRL in endocrinology (24). The present study also reported a positive protective effect of prescription aripiprazole upon PRL levels in chronic long-term hospitalized patients with schizophrenia. However, this protective effect was not reflected in the subgroup including patients with comorbid DM. We have yet to come across any report on the interaction between aripiprazole, PRL, and DM. This does not exclude the possibility of anomalous results as a result of insufficient statistical strength, due to a lack of cases in the subgroup prescribing aripiprazole (12, 8.16%). In the future, we will investigate whether the abnormal metabolic indicators, insulin resistance, and PRL levels of co-DM patients have a deeper unexplored influence mechanism on the protective effect of aripiprazole on hyper-prolactin.

The fact that the subgroups with co-DM had lower PRL levels was another significant finding in this study. Here, it must be emphasized that metformin is not only the basic drug and first-choice drug for diabetes treatment (25), but also an effective drug against HPRL caused by antipsychotic drugs (23). Therefore, prescription metformin to people with schizophrenia and diabetes is a choice that can help patients in many different ways. The fact that metformin protects against HPRL and has a dose-dependent impact is also a crucial finding. An expert consensus also agrees that high-dose metformin is more effective in improving antipsychotic-induced HPRL (26). Researchers also believe that this pharmacological property of metformin is related to its ability to improve endogenous dopaminomimetic activity (23, 27). Furthermore, there are few studies where researchers demonstrated the connection between PRL and diabetes. One study found that PRL was inversely related to glucose levels in young, healthy subjects (28). Another study reported an inverse relationship between serum PRL levels and insulin in men who were overweight or obese (29). However, the mechanism underlying this has not been clearly articulated. Our findings indicate that patients with co-morbid DM had more severe psychiatric symptoms and lower PRL levels; whereas high levels of PRL had a protective effect on the brain and reduced the psychiatric symptoms of the patients (17, 30). This may be another reasonable explanation for the lower PRL level in the clinical subgroup of diabetes.

Differences due to gender were also evident in PRL levels in schizophrenia patients, and female was at greater risk of HPRL. This risk persists in the subgroup of co-diabetes. A study of new-onset drug-naive schizophrenia reported that women had higher mean PRL levels than men (31). Another study on patients with first-episode non-affective psychosis reported smaller changes in mean PRL level in men than in women (32). However, higher PRL levels imply a wider range of PRL-related adverse effects (33), severe abnormalities in glucose and lipid metabolism (34), and significant impulsive behavior (35). In the present study, the psychiatric symptoms were found to be more severe in the group with DM. To summarize, female may be at a disadvantage in maintaining low levels of PRL throughout the disease. We, therefore, suggest that this phenomenon be specifically taken into account as a risk factor for HPRL when prescribing treatment medications for female patients. There are also some shortcomings in this study. Since the enrolled sample comprised chronic patients who were hospitalized for a long time, they were prescribed more complicated medications, including antipsychotics and hypoglycemic drugs. Hence, only qualitative analysis was performed when carrying out statistical analysis, and the effect of medication dose on statistical results is thus not known. When performing subgroup analysis, the sample size of the diabetes subgroup prescribed with aripiprazole was small, which may affect the statistical efficacy. In the future, more stringent inclusion criteria will be employed, and the sample size will be further expanded to compensate for these deficiencies.

## Conclusion

In short, co-morbid DM is associated with extreme residual psychiatric symptoms and more severe adverse effects (including metabolic abnormalities and HPRL) in patients with long-term hospitalization due to chronic schizophrenia. Aripiprazole is a protective factor for HPRL in long-term hospitalized patients and female is a risk factor, while metformin is beneficial in reducing PRL levels in patients with co-morbid DM. More aggressive and effective interventions are needed for fighting adverse drug reactions in women and patients with co-morbid DM.

## Data availability statement

The original contributions presented in this study are included in the article/supplementary material, further inquiries can be directed to the corresponding author/s.

## Ethics statement

This study was reviewed and approved by the Ethics Committee of Wuhan Mental Health Center. All subject guardians knew about this study and signed informed consent.

## Author contributions

JM and YL made substantial contributions to the conception and design of the study. JZ drafted the manuscript. HW and SH polished and re-edited the language and logic of the manuscript. YZ and XL were responsible for setting up and complementing and modifying the contents of the manuscript. JM gave final approval for the version to be published. All authors contributed to the article and approved the submitted version.
